# GPR30 agonist G1 combined with hypothermia alleviates cognitive impairment and anxiety‐like behavior after subarachnoid hemorrhage in rats

**DOI:** 10.1002/brb3.3204

**Published:** 2023-08-07

**Authors:** Jun Peng, Yang He, Jun He, Ji‐kun Zhang, Zheng‐tao Yu, Ying Xia

**Affiliations:** ^1^ Department of neurosurgery Haikou Affiliated Hospital of Central South University Xiangya School of Medicine Haikou Hainan China; ^2^ Department of Neurology Hainan Medical College First Affiliated Hospital Haikou Hainan China

**Keywords:** anxiety‐like behavior, cognitive impairment, GPR30 agonist G1, hypothermia therapy, rats, subarachnoid hemorrhage

## Abstract

**Introduction:**

This study aimed to investigate the treatment effect of G protein‐coupled receptor 30 (GPR30) agonist G1 combined with hypothermia (HT) on cognitive impairment and anxiety‐like behavior after subarachnoid hemorrhage (SAH) in rats.

**Methods:**

Fifty male rats were randomly assigned to one of five groups: Sham group, SAH group, SAH + G1 group, SAH + HT group, and SAH + G1 + HT group. The SAH rat model was established by modified endovascular puncture in all groups except the Sham group. Neurological function after the operation was assessed by Garcia scoring. The degree of rat cerebral edema was determined using dry‐wet weighing method on the 28th day after operation. Moreover, the behavioral test was performed on rats on the 4th and 28th days after operation.

**Results:**

Compared with Sham group, the Garcia score of each SAH rat model group decreased significantly on the first day and thereafter increased gradually. However, the recovery rate of each treatment group was higher than the SAH group (no treatment), and the Garcia score of SAH + G1 + HT group was much higher than the SAH group on the seventh day after operation. In addition, each treatment group could obviously reduce the cerebral edema degree of SAH rats, among which rats in SAH + G1 + HT group had lower cerebral edema degree than SAH + G1 group and SAH + HT group. Behavioral test results showed that the combination of GPR30 agonist G1 and HT markedly improved the learning and memory ability of SAH rats, alleviated their anxiety‐ and emotion‐related behavior, and enhanced their social interaction.

**Conclusion:**

GPR30 agonist G1 combined with HT reduces cognitive impairment and anxiety‐like behavior in rats with SAH.

## Graphical abstract

investigating the treatment effect of G protein‐coupled receptor 30 (GPR30) agonist G1 combined with hypothermia (HT) on the cognitive impairment and anxiety‐like behavior after subarachnoid hemorrhage (SAH) in rats.

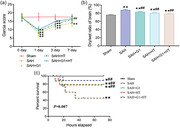

## 1 INTRODUCTION

Subarachnoid hemorrhage (SAH) refers to a clinical syndrome caused by a leakage of blood into the spinal subarachnoid space after rupture of blood vessels at the base or surface of the brain. It is a severe and common disease accounting for about 10% of factors causing acute stroke (Mahmoud & Mason, 2015). It is reported that about 80% of SAH is caused by rupture of aneurysm, and the remainder by vascular malformation and vasculitis (Chen et al., 2017). The early manifestations of SAH include sudden severe headache, nausea, vomiting, and meningeal irritation, with or without the presence of focal neurologic signs. In advanced patients, poor prognosis such as hemiplegia, aphasia, cognitive impairment, and even death often occur, which is mainly associated with early brain injury and delayed brain injury caused by SAH (Jang et al., 2017). As an important feature of early brain injury, cerebral edema is an independent risk factor causing death and poor prognosis after developing SAH. Cerebral nerve injury after SAH, the main obstacle to the recovery of SAH patients, may cause cognitive ability decline and mental disorders such as anxiety and depression, and make it difficult for patients to return to work or live a functional life (Lawton & Vates, 2017).

Currently, superselective intra‐arterial embolization or craniotomy clipping is the preferred treatment for SAH. However, the two treatments have side effects, and the effect of neurological function recovery after treatment still needs to be improved (Cruz et al., 2013). Mild hypothermia (HT) nursing is a method to physically control the body temperature of the patient and inhibit vasogenic edema and bleeding, so as to regulate intracellular calcium ion aggregation and excitatory neurotransmitter release rate, maintain normal brain metabolism, and thus protect the neurological function (Thomé et al., 2005). However, the effect of mild HT nursing alone is not enough to achieve the expectation. Therefore, the need for more effective treatment for SAH is urgent. G protein‐coupled receptor 30 (GPR30), a novel membranous estrogen receptor, can play the role of transcriptional regulation indirectly through the second messenger system (Bologa et al., 2006). The studies indicated that GPR30 plays a vital role in the regulation of neurological functions, such as neuroprotection, synaptic regulation, improvement of learning and memory ability, and neurogenesis (Kumar et al., 2020). G1, the specific agonist of GPR30, is effective in treating brain disease due to its multiple functions. Studies have found that G1 can alleviate neuronal apoptosis in male rats with SAH by activating src/EGFR/stat3/signaling pathway, thereby alleviating the brain injury (Peng et al., 2019). It follows then that G1 may provide a prospective treatment strategy for SAH patients. However, there are still no studies reporting whether the combination of GPR30 agonist G1 and HT can improve the cognitive impairment and anxiety‐like behavior of SAH patients or not. Therefore, this study was proposed to explore the application effect of G1 or HT alone or the combination of the two on cognitive impairment and anxiety‐like behavior of SAH patients by establishing the SAH rat model, in order to provide a new strategy for the clinical treatment of SAH.

## 2 MATERIALS AND METHODS

### 2.1 Establishment and grouping of rat SAH model

Totally 50 male standard deviation (SD) rats (age: 6–8 weeks; weight: 180–220 g) were purchased from Shanghai Model Organisms Center, Inc. All rats were treated under the protocol approved by the Institutional Animal Care and Use Committee and allowed for 1‐week adaptation before the experiment. All experiments in this study were approved by the Animal Ethics Committee of Haikou Affiliated Hospital of Central South University Xiangya School of Medicine (2022–200).

The rats were randomly divided into five groups: Sham group (*n* = 10), SAH group (*n* = 10), SAH + G1 group (*n* = 10), SAH + HT group (*n* = 10), and SAH + G1 + HT group (*n* = 10). The treatment for each group is as follows. SAH group: SAH model was established by modified endovascular puncture based on the modified modeling method of Guo et al. (2016). Specifically, the rats were anesthetized by intraperitoneal injection of 1% pentobarbital sodium (Boster Biological Technology Co. Ltd) at a dose of 50 mg/kg. After the anesthesia, the rats were subjected to exposure of the common carotid artery, external carotid artery, and internal carotid artery, and then a nylon thread was used to run through the internal carotid artery. When the nylon thread reached the intracranial bifurcation, it was pushed further to 3 mm. After the puncturation of the blood vessel and the apical stitch, the blood flow was restored. SAH + G1 group: GPR30 agonist G1 (300 μg/kg, Gayman Company) was intravenously injected 1 h after the establishment of SAH model. SAH + HT group: The rats were intermittently placed in a 5°C cryogenic box for 12 h after the establishment of SAH model and then fed for 12 h. The rectal temperature was kept at 32–34°C. SAH + G1 + HT group: GPR30 agonist G1 (300 μg/kg) was intravenously injected 1 h after the establishment of SAH model, and the rest of operations were the same as the SAH + HT group. Sham group: After anesthesia of the rats, a midline incision of the neck was performed to expose the common carotid artery, external carotid artery, and internal carotid artery. A nylon thread ran through the internal carotid artery and was pushed forward until it reached the neck. Notably, vascular puncture was not performed. Behavioral tests of the rats were performed on the 4th and 28th days after the operation. Calculate the mortality rate of rats, and subsequently take the brain tissue of rats.

### 2.2 Garcia neurological function score

The neurological function of rats in each group was evaluated using a Garcia scale (Garcia et al., 1995) before the operation and on the first, third, and seventh days after the operation. This scale items included rats’ voluntary movement, activity symmetry of limbs, stretching ability of forepaws, ability to climb and grasp the iron cage, physical sense reaction, and reaction to beard touching. Rats with normal functions had the highest score (18 points), and those with the most severely impaired functions had the lowest score (3 points).

### 2.3 Determination of cerebral edema degree

Brain tissue was rapidly separated on ice from rats after deep anesthesia by intraperitoneal injection, and left, and right cerebral hemispheres were taken. The wet weight of the brain tissue was measured by electronic analytical balance, and the tissue was baked in an incubator for 48 h. The tissue specimens were cooled for 10 min after being taken out of the incubator. Then its dry weight was weighed by the electronic analytic balance. The water content of the brain tissue was calculated using the dry‐wet weighing method, which is an index to evaluate the degree of cerebral edema (Li et al., 2015).

### 2.4 Behavioral tests of rats

Morris water maze test (MWM): A 4‐day training experiment was performed to evaluate the spatial learning and memory ability of rats in each group. The escape latency (1–4 days), number of entries in the target quadrant, the time spent in the target quadrant, and the average swimming speed of the rats were recorded. The worse the cognitive function of experimental rats, the longer the escape latency and swimming distance, the less the number of entries in the target quadrant, and the shorter time spent in the target quadrant (Bromley‐Brits et al., 2011).

Novel object recognition test (NORT): The exploration time of rats in each group toward familiar objects (TF) and novel objects (TN) during the test phase was recorded. If the rat's cognitive ability was bad, there was no difference between the time of TN and that of TF. If the rat's cognitive ability was normal, the time spent in exploring TN would be longer than that of exploring TF. The recognition index (RI) of exploring novel objects was calculated as RI = TN/(TF + TN) (Leger et al., 2013).

Open field test (OFT): Rats were placed in the center zone of a black square device (100 cm × 100 cm × 40 cm). The free activity of the rats within 5 min was filmed by a camera and recorded by SMART software. Before the test, the rats were transported to the test room and allowed to adapt for 15 min. The noise in the room should be kept at 30 decibels and the light at 60 W. The rats were placed in the center of the open field gently and allowed to explore for 5 min. The total traveled distance and time in the central zone were recorded. The total traveled distance reflected the movement of the rat, whereas the time in the central zone reflected the anxiety of the rat. The horizontal traveled distance and time in the central zone of depressed rat decreased significantly (Gould et al., 2009).

Elevated plus maze (EPM): The EPM consisted of two open shelves (50 cm × 10 cm × 40 cm), a central zone (5 cm × 5 cm), and two closed shelves (50 cm × 10 cm × 40 cm) (CWE company). The rats were placed at the center of EMP and their tracks in 5 min were recorded by the camera and the software. Before the test, the rats were transported into the test room and allowed to adapt for 15 min. The noise in the room should be kept at 30 decibels. The maze was positioned 100 cm above the floor and the experiment was carried on in an environment as dark as possible. The rats were then placed at the center platform and the open arm entries of the rats and total time spent on open arms were recorded. The EPM reflected the anxiety of the rats. After receiving antianxiety drugs, the rats had an obvious increase in the number of open arm entries of the rats and the total time spent in open arm (Kraeuter et al., 2019).

Three‐chamber paradigm test: An empty metal cage (E) was placed in the rightmost chamber and a strange rat of the same species (S) in a mental cage was placed in the leftmost chamber. The tested rat (placed in the center chamber) was allowed to move freely among the three rooms. The time the tested rat spent in the chambers on either side was recorded respectively to evaluate its sociability. Normal rats would spend more time in the S chamber (Moy et al., 2004).

Reciprocal social interaction test: A tested rat and an S rat were simultaneously placed into a box (40 cm × 40 cm × 30 cm) and allowed to explore each other freely for 10 min. The number of bouts, including crawl under and crawl over (push‐crawl), nose‐to‐nose, anogenital investigation (nose‐to‐anogenital), and self‐grooming behaviors, were recorded (Matsuda et al., 2016).

### 2.5 Nissl staining

Paraffin‐embedded rat hippocampal tissue sections were dewaxed and stained with 1% toluidine blue solution (Solarbio) for 10 min. Then, after washing with distilled water, Nissl differentiation solution was added dropwise to the tissue sections for 2–4 min. Subsequently, 95% ethanol was added for rapid differentiation, and after the Nissl changed to purple and the other tissues became colorless. The sections were dehydrated with anhydrous ethanol (Solarbio), transparent with xylene (Solarbio), and finally sealed with neutral glue (Solarbio) to seal the sections. Images were captured using an optical microscope (Olympus).

### 2.6 Western blot

Follow the instructions provided by the manufacturer of the protein extraction kit (Beyotime Biotech Inc.) to extract proteins from hippocampal tissue. The protein was separated by 10% sodium dodecyl sulfate polyacrylamide gel electrophoresis (Bio Rad laboratory), and then the protein in the gel was transferred to the polyvinylidene fluoride membrane. Seal the membrane with 5% skim milk for 2 h. Then, the membrane was mixed with GPR30 (1:1000, Abcam) and β‐incubate overnight with actin (1:5000, Abcam). Subsequently, incubate at room temperature with corresponding goat anti mouse and goat anti rabbit (1:5000, Abcam) for 2 h. Finally, the Tanon‐5200 chemiluminescence imaging system was used to detect protein bands, and Image software was used to quantify the gray scale of protein bands.

### 2.7 Statistical analysis

SPSS 21.0 software was used for statistical analysis. *t*‐Test was used for comparison between two groups, whereas one‐way analysis of variance for paired comparison among multiple groups. The results were expressed by mean ± SD. *p* < .05 was considered the criterion for significant difference.

## 3 RESULTS

### 3.1 G1 combined with hypothermia improves the neurological function and alleviates cerebral edema degree of rats with subarachnoid hemorrhage

**FIGURE 1:**
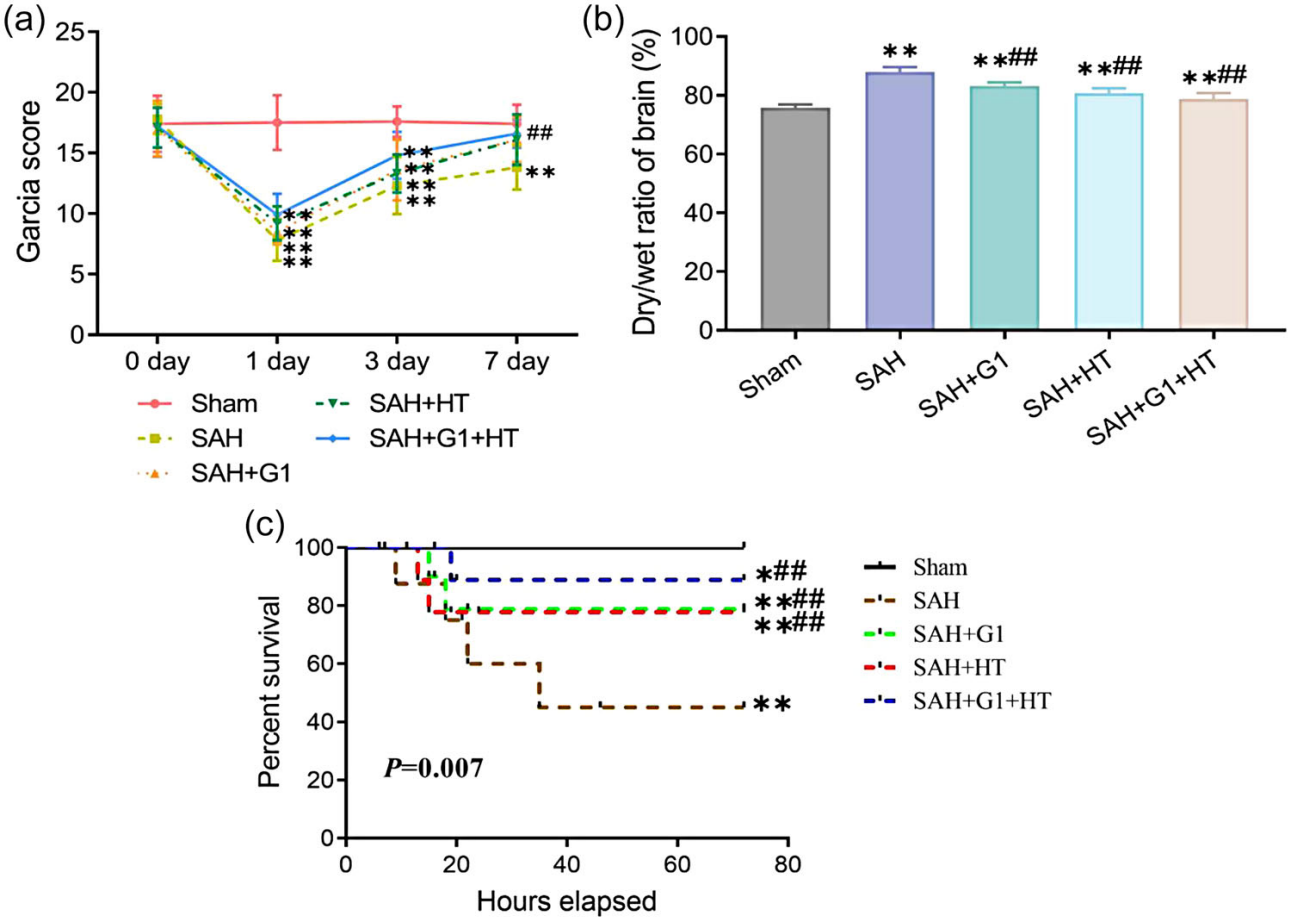
G1 combined with hypothermia improves the neurological function and alleviates cerebral edema degree of subarachnoid hemorrhage (SAH) rats. (a) The neurological function of rats in each group before operation and the first, third, and seventh days after operation evaluated by Garcia score scale. (b) The cerebral edema degree of rats in each group measured by dry‐wet weighing method 28th day after operation. (c) The mortality rates of rats in Sham group, SAH group, SAH + G1 group, SAH + hypothermia (HT) group, and SAH + G1 + HT group within 72 h were recorded, and Kaplan–Meier survival curves were plotted. ^**^ *p* < .01, versus Sham group; ^##^ *p* < .01, versus SAH group (*n* = 6).

The results indicated that there was no significant difference in the Garcia score of rats in each group before the operation (*p* > .05). On the first day after the operation, the Garcia score of rats in each SAH model group decreased significantly (*p* < .05) and increased gradually thereafter. The recovery rates of all treatment groups were higher than in the SAH group. On the seventh day after the operation, the Garcia score of the SAH group decreased markedly compared to the Sham group (*p* < .05), but there was no obvious difference in each treatment group (*p* > .05). The Garcia score of the SAH + G1 + HT group was notably higher than the SAH group (Figure 1a). In addition, the results of the dry/wet ratio of brain tissue revealed that the cerebral edema degree of rats in each SAH model group was much higher than the Sham group (*p* < .05). Compared to the SAH group, each treatment group displayed a significant decline in the cerebral edema degree of rats (*p* < .05). However, the cerebral edema degree of the SAH + G1 + HT group was lower than the SAH + G1 group and the SAH + HT group (*p* < .05) (Figure 1b). In addition, we analyzed the survival of rats during the experimental period, and the results showed that there was no rat death in the Sham group; the mortality rate of SAH rats was significantly increased compared with the Sham group (*p* < .05); and the mortality rate of SAH + G1, SAH + HT, and SAH + G1 + HT rats was significantly decreased compared with the SAH group (*p* < .05). Meanwhile, the mortality rate of rats in the SAH + G1 group and SAH + HT group was slightly higher than that in the SAH + G1 + HT group, but the difference was not statistically significant (Figure 1c) (*p* > .05).

### 3.2 G1 combined with hypothermia improves the learning and memory ability of rats with subarachnoid hemorrhage

**FIGURE 2:**
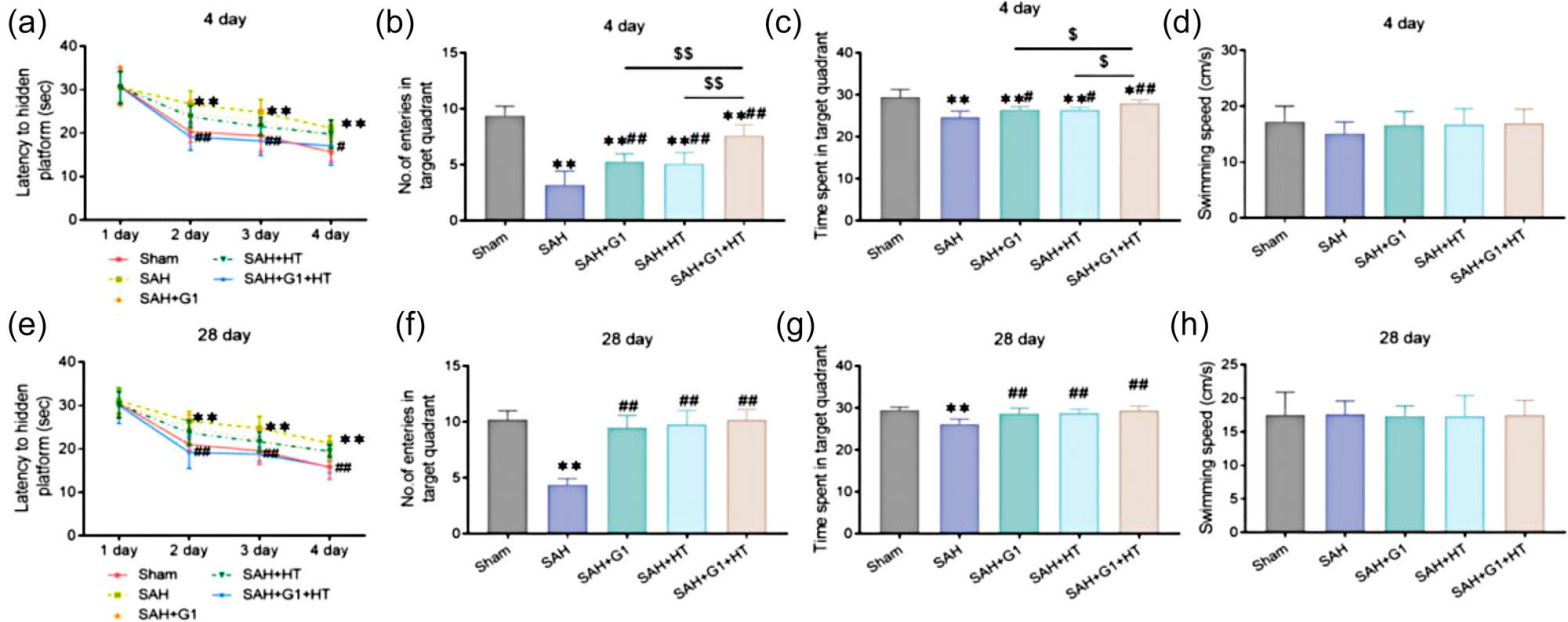
G1 combined with hypothermia improves the learning and memory ability of subarachnoid hemorrhage (SAH) rats. (a–d) Morris water maze test (MWM) results (the fourth day after operation) of escape latency (1–4 days) (a), number of entries in target quadrant (b), time spent in target quadrant (c), and average swimming speed (d). (E–H) MWM results (the 28th day after operation) of on the, escape latency (1–4 days) (e), number of entries in target quadrant (f), time spent in target quadrant (g), and average swimming speed (h). ^**^ *p* < .01, versus Sham group; ^##^ *p* < .01, ^#^ *p* < .05, versus SAH group; ^$$^ *p* < .01, ^$^ *p* < .05, versus SAH + G1 + hypothermia (HT) group (*n* = 6).

MWM results showed that on the fourth day after operation, the 3‐day escape latency of rats after training was obviously prolonged in the SAH group (*p* < .01). The number of entries in the target quadrant and the time spent in the target quadrant was markedly reduced in each SAH model group (*p* < .05). However, the SAH + G1 + HT group had an obvious shorter escape latency (*p* < .01), as well as an obvious larger number of entries in the target quadrant and longer time spent in the target quadrant of each treatment group than the SAH group (*p* < .05). The number of entries in the target quadrant and the time spent in the target quadrant of the SAH + G1 + HT group was markedly raised relative to the SAH + G1 group and SAH + HT group (*p* < .05). Notably, there was no significant difference in average swimming speed among these groups (*p* > .05) (Figure 2a–d).

On the 28th day after operation, the 3‐day escape latency of rats after training was significantly prolonged in the SAH group relative to the Sham group (*p* < .01). The number of entries in target quadrant and time spent in the target quadrant markedly decreased in the SAH group relative to the Sham group (*p* < .05). Compared to the SAH group, the SAH + G1 + HT group exhibited an obvious decrease in escape latency (*p* < .01). A notable increase in the number of entries in the target quadrant and time spent in the target quadrant was found in each treatment group in comparison with the SAH group (*p* < .05). Likewise, there was no significant difference in average swimming speed among these groups (*p* > .05) (Figure 2e–h).

3.3

**FIGURE 3:**
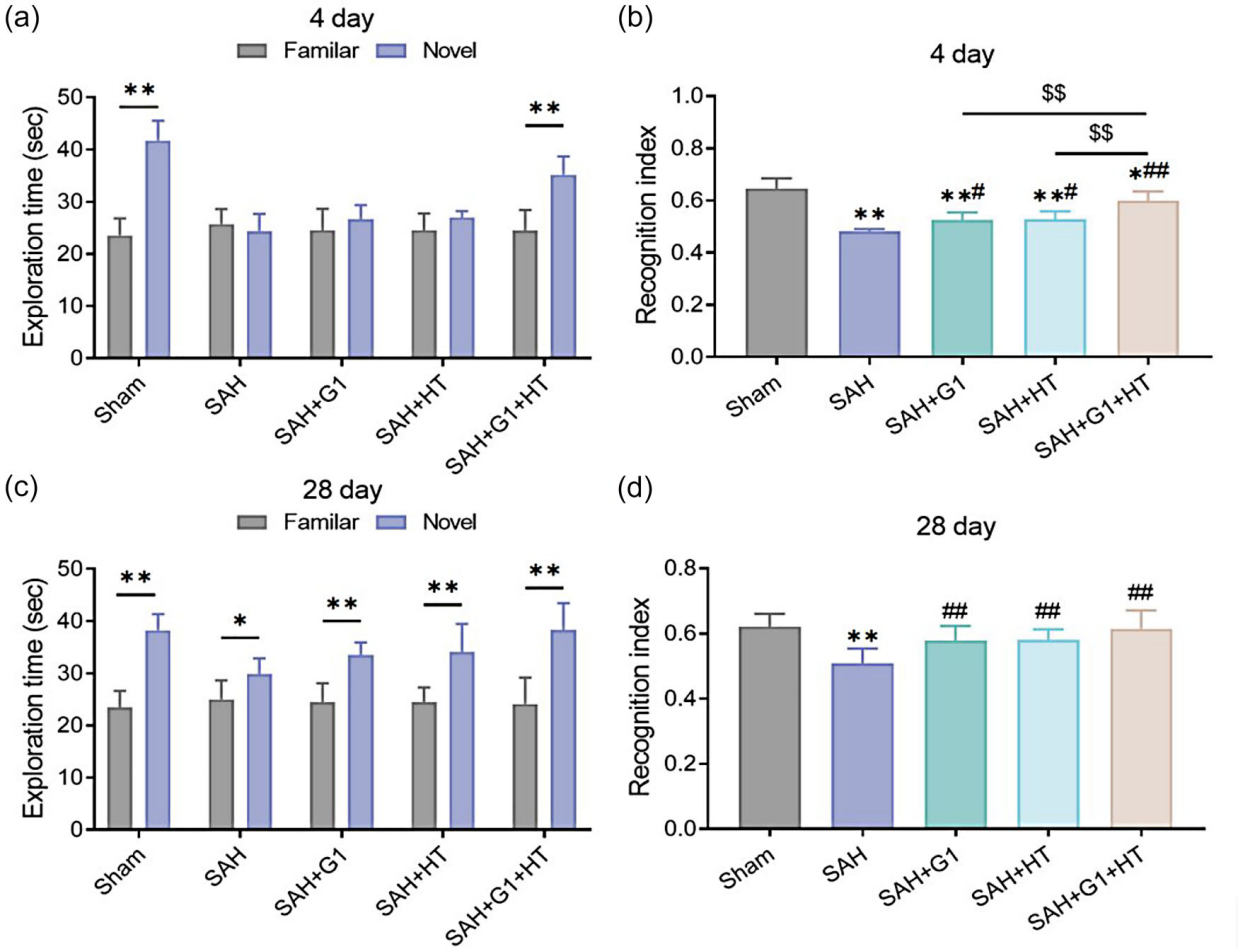
G1 combined with hypothermia improves the learning and memory ability of subarachnoid hemorrhage (SAH) rats. (a and b) Novel object recognition test (NORT) results (the fourth day after operation) of the exploration time (a) of rats in each group toward familiar objects (TF) and novel objects (TN) during the testing phase and of recognition index (RI) (b) calculated as RI = TN/(TF + TN). (C and D) NORT results (the 28th day after operation) of the exploration time (c) of rats in each group toward TF and TN during the testing phase and of RI (d) calculated as RI = TN/(TF + TN). ^**^ *p* < .01, versus Sham group, ^##^ *p* < .01, versus SAH group, ^$$^ *p* < .01, versus SAH + G1 + hypothermia (HT) group (*n* = 6).

In additional, NORT results showed that, on the fourth day after operation, rats in the Sham group spent observably more time on exploring TN than on TF (*p* < .01), and had much higher RI than other groups (*p* < .01). There was no significant difference between the time spent on exploring TN and on exploring TF by rats in the SAH group, the SAH + G1 group, and the SAH + HT group. Rats in the SAH + G1 + HT group spent considerably more time on exploring TN than on TF (*p* < .01), but the time spent on exploring TN was less than the Sham group. Compared to that of the SAH group, RI of each treatment group was notably higher (*p* < .05). By the way, RI of the SAH + G1 + HT group was markedly higher than that of the SAH + G1 group and the SAH + HT group (*p* < .05) (Figure 3a,b).

As for the 28th day after the operation, rats in each group spent more time on TN than on TF. However, the time rats in the SAH group spent on exploring TN was quite shorter than the other groups, and the time rats in the SAH + G1 + HT group spent on exploring TN was longer than the SAH + G1 group and the SAH + HT group (*p* < .05). Compared to the Sham group, RI of rats in the SAH group declined significantly (*p* < .05), whereas compared to the SAH group, RI of each treatment group increased markedly (*p* < .05) (Figure 3c,d).

### 3.4 G1 combined with hypothermia reduces anxiety‐ and emotion‐related behavior of rats with subarachnoid hemorrhage

**FIGURE 4:**
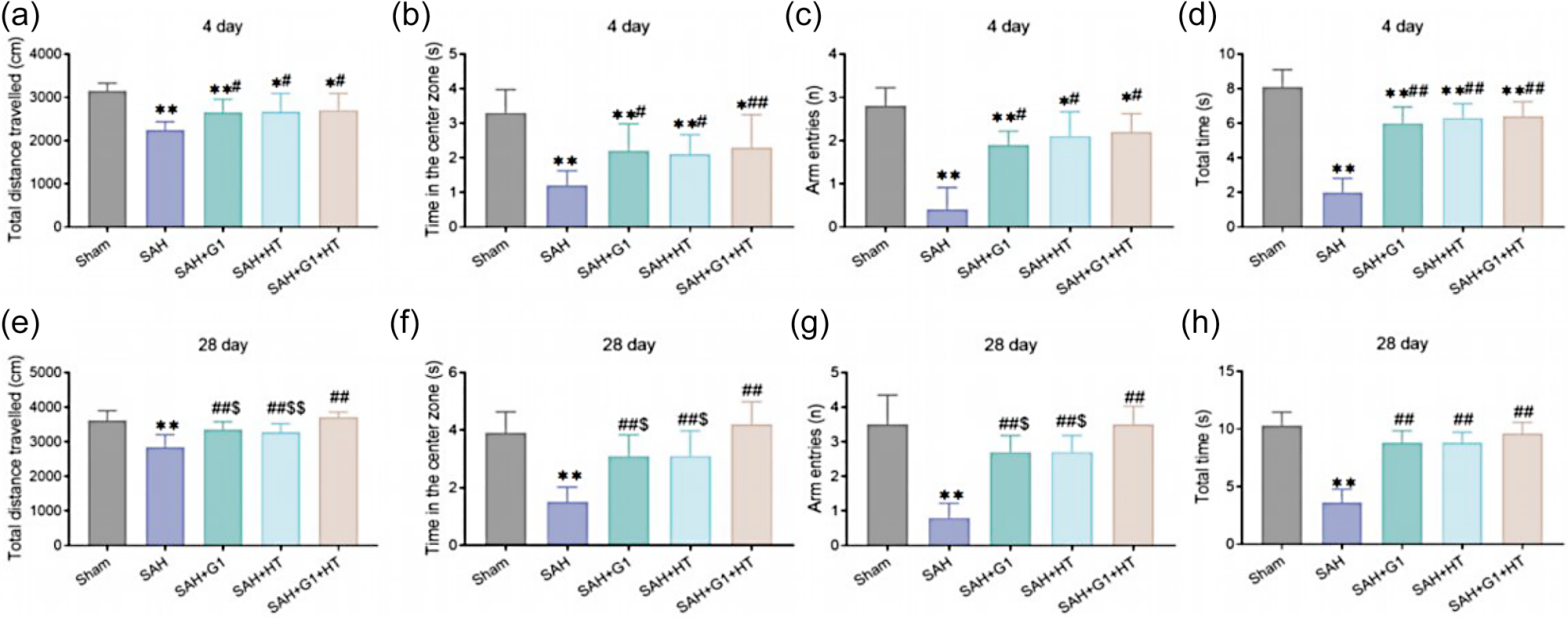
G1 combined with hypothermia reduces anxiety‐ and emotion‐related behavior of subarachnoid hemorrhage (SAH) rats. (a–d) Open field test (OFT) results (the fourth day after operation) of total traveled distance (a) and time in the central zone (b) of rats. Elevated plus maze (EPM) results (the fourth day after operation) of arm entries (c) and total time spent in open arm (d) of rats. (e–h) OFT results (the 28th day after operation) of total traveled distance (e) and time in the central zone (f) of rats. EPM results (the 28th day after operation) of arm entries (g) and total time spent in open arm (h) of rats. ^**^ *p* < .01, ^*^ *p* < .05, versus Sham group, ^##^ *p* < .01, ^#^ *p* < .05, versus SAH group, ^$$^ *p* < .01, ^$^ *p* < .05, versus SAH + G1 + hypothermia (HT) group (*n* = 6).

As revealed by the tests performed on the fourth day after the operation, rats in each SAH model group showed a significant decline in the total traveled distance, time in the central zone, arm entries, and total time spent in the open arm of rats (*p* < .01); compared to the SAH group, the total traveled distance, time in the central zone, arm entries, and total time spent in the open arm of rats in each treatment group increased markedly (*p* < .01); and compared to the SAH + G1 group and the SAH + HT group, the total traveled distance, time in the central zone, arm entries, and total time spent in open arm of the SAH + G1 + HT group were slightly higher, but no significant difference existed (*p* > .05) (Figure 4a–d).

On the 28th day after the operation, the total traveled distance, time in the central zone, arm entries, and total time spent in the open arm of SAH rats noticeably decreased relative to the Sham group (*p* < .01). Compared to the SAH group, the total traveled distance, time in the central zone, arm entries, and total time spent in the open arm of each treatment group markedly increased (*p* < .01). The SAH + G1 + HT group showed obviously higher total traveled distance, time in the central zone, arm entries, and total time spent in open arm than the SAH + G1 group and the SAH + HT group (*p* < .01) (Figure 4e–h).

### 3.5 G1 combined with hypothermia enhances the social interaction of rats with subarachnoid hemorrhage

**FIGURE 5:**
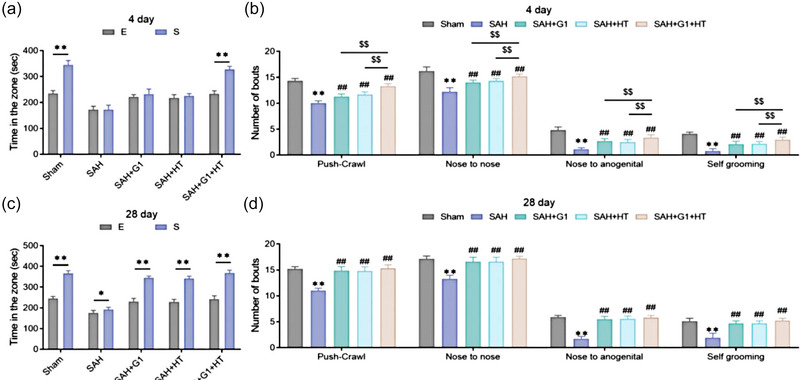
G1 combined with hypothermia enhances the social interaction of subarachnoid hemorrhage (SAH) rats. (a and b) Three‐chamber paradigm test results (the fourth day after operation) of the time rats spent in two chambers (a). Reciprocal social interaction test results (the fourth day after operation) of the number of push‐crawl, nose‐to‐nose, nose‐to‐anogenital, and self‐grooming bouts (b). (c and d) Three‐chamber paradigm test results (the 28th day after operation) of the time rats spent in two chambers (c). Reciprocal social interaction test results (the 28th day after operation) of the number of push‐crawl, nose‐to‐nose, nose‐to‐anogenital, and self‐grooming bouts (d). ^**^ *p* < .01, versus Sham group, ^##^ *p* < .01, versus SAH group, ^$$^ *p* < .01, versus SAH + G1 + hypothermia (HT) group (*n* = 6).

In order to assess the sociability of SAH rats, we carried out a three‐chamber paradigm test and a reciprocal social interaction test. It was found that on the fourth day after the operation, rats of the Sham group and the SAH + G1 + HT group spent more time in the S chamber than in the E chamber (*p* < .01), but there was no significant difference between the time spent on S chamber than on E chamber of rats in the SAH, the SAH + G1 group, and the SAH + HT group (*p* > .05); compared to the Sham group, the number of reciprocal social interaction bouts of SAH rats significantly declined (*p* < .01); compared to the SAH group, the number of reciprocal social interaction bouts of rats in each treatment group increased markedly (*p* < .05); the number of reciprocal social interaction bouts of rats in the SAH + G1 + HT group was obviously larger than the SAH + G1 group and the SAH + HT group (*p* < .05) (Figure 5a,b).

On the 28th day after the operation, the time spent on the S chamber and the reciprocal social interaction bouts of rats in the SAH group were much less than the Sham group (*p* < .01). Nevertheless, those in each treatment group were markedly higher than the SAH group (*p* < .01). The time spent on the S chamber and the reciprocal social interaction bouts of rats in the SAH + G1 + HT group were slightly mounted relative to the SAH + G1 group and the SAH + HT group, but no significant difference was displayed between them (*p* > .05), as well as between the Sham group and the SAH + G1 + HT group (*p* > .05) (Figure 5c,d).

### 3.6 G1 combined with hypothermia treatment reduces neuronal damage in hippocampal tissue of SAH rats

**FIGURE 6:**
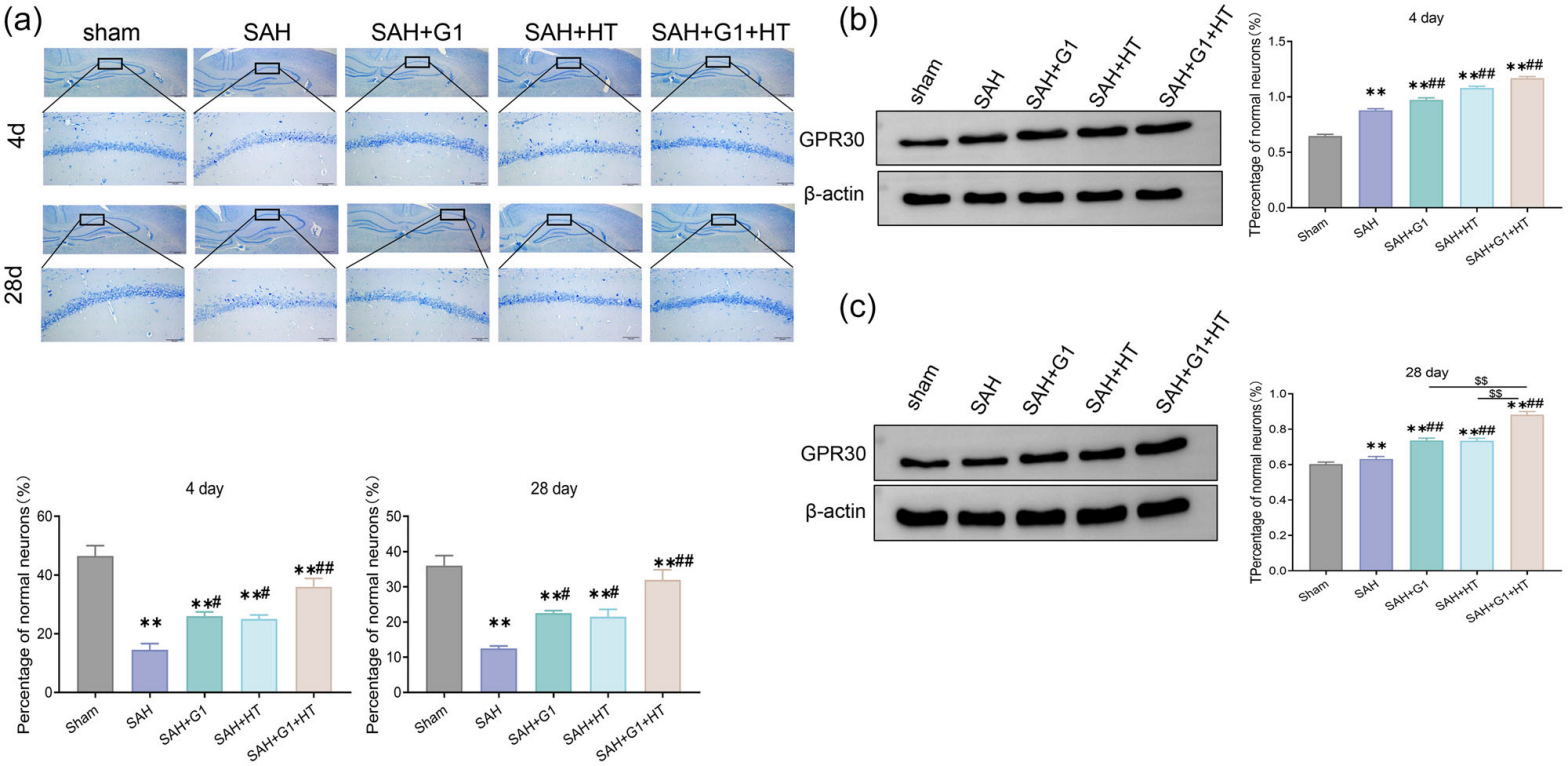
G1 combined with hypothermia treatment reduces neuronal damage in hippocampal tissue of subarachnoid hemorrhage (SAH) rats. (b and a) Nissl staining was used to detect the morphological changes of hippocampal CA1 neurons in Sham group, SAH group, SAH + G1 group, SAH + hypothermia (HT) group, and SAH + G1 + HT group rats on 4th and 28th days. (B/C) Western blot was used to detect the protein levels of G protein‐coupled receptor 30 (GPR30) in the brain tissues of rats in Sham group, SAH group, SAH + G1 group, SAH + HT group, and SAH + G1 + HT group on 4th and 28th days. ^**^ *p* < .01, versus Sham group, ^##^ *p* < .01, versus SAH group (*n* = 6).

In the present study, the histopathological changes of the hippocampus of SAH rats were detected by Nissl staining, and the results showed that at 4 and 28 days, no significant changes were found in the morphological changes of neurons in the CA1 area of the hippocampus of the Sham group rats, with clear neuronal demarcation, abundant cytoplasm, and dome‐shaped nuclei. At 4 days, compared with the Sham group, different degrees of neuronal damage and loss in hippocampal tissues were seen in SAH rats after different treatments, with shrunken cell bodies, condensed nuclei, concentrated and deeply stained nuclei, and cytoplasm, and the ratio of normal neurons was significantly reduced; compared with the SAH group (*p* < .01), G1 alone or HT or G1 combined with HT treatment significantly improved the pathology of hippocampal tissues in SAH rats damage and increased the ratio of normal neurons (*p* < .01). In addition, the ratio of normal neurons in the SAH + G1 + HT group was significantly higher than that in the SAH + G1 and SAH + HT groups (*p* < .01). At 28 days, different degrees of neuronal loss were still visible in the hippocampal tissues of SAH, SAH + G1, and SAH + HT groups, and a small amount of neuronal loss occurred in the SAH + G1 + HT group (*p* < .01) (Figure 6a). In addition, we also examined the protein levels of GPR30 at 4 and 28 days, and the results showed that at 4 and 28 days, there was no significant difference in the protein levels of GPR30 in the brain tissues of rats in the Sham group (*p* > .05). At 4 and 28 days, the protein levels of GPR30 were significantly higher in SAH rats compared with the Sham group; compared with the SAH group, the protein levels of GPR30 were significantly higher in the SAH + G1 group, SAH + HT group, and SAH + G1 + HT group (*p* < .01), and the protein levels of GPR30 in hippocampal tissues of SAH rats after G1 combined with HT treatment were significantly higher than those in the SAH + G1 group and SAH + HT group (*p* < .01) (Figure 6b,c).

## 4 DISCUSSION

SAH is a cerebral hemorrhage condition that seriously damages the brain and causes cognitive impairment (Mahmoud & Mason, 2015). Clinical emphasis has been directed toward saving life and lowering the mortality and mutilation rate, but not toward cognitive impairment after SAH (Cornea et al., 2022). Based on foreign relevant statistics, at least more than 60% of SAH patients have cognitive impairment at various degrees, involving impairment in multiple cognitive domains, such as attention, memory, orientation, calculability, as well as analytical, comprehensive, judgment and executive abilities (Al‐Khindi et al., 2010). Among them, memory, executive ability, and language are most commonly involved. The cognitive impairment associated with these domains usually occurs within 3 months of onset and even lasts up to 5 or more years after onset (Proust et al., 2009), which poses tremendous difficulties in both work and the life of the patient. Therefore, it is crucial to seek effective drugs or treatment that can alleviate cognitive impairment after SAH.

GPR30, a novel estrogen transmembrane receptor, can regulate rapid non‐genomic estrogen signal transduction (Revankar et al., 2005). It is mainly expressed in brain areas of the central nervous system, such as the hippocampus, cortex, and striatum (Hammond & Gibbs, 2011). Previous studies have shown that the neuroprotective effect of GPR30 agonist G1 on hippocampus and striatum before CA/CPR was similar to that of estrogen 2, indicating that this agonist has the dual effect of estrogen. Accordingly, activation of GPR30 has clinical value in the protecting brain tissue, and GPR30 also provides new ideas for estrogen replacement therapy in cerebral ischemia (Prossnitz et al., 2008). In addition, G1 can reduce the infarction size of hippocampus and striatum after cerebral ischemia (Kuiper et al., 1996). In a global brain ischemia model induced by vascular occlusion, G1 was demonstrated to reduce the neuronal mortality in the hippocampal CA1 region after cerebral ischemia and improve the cognitive impairment of neurons (Zhang et al., 2010). Our study revealed that Garcia scores of rats in all SAH model groups declined significantly on the first day after the operation, and the cerebral edema degree decreased markedly and increased gradually thereafter. However, the SAH + HT group, the SAH + G1 group, and the SAH + G1 + HT group had higher recovery rates but lower cerebral edema degree than the SAH group. Garcia score and cerebral edema degree of the SAH + G1 + HT group were better than the SAH + HT group and the SAH + G1 group. The above suggested that G1 combined with HT improved the neurological function of SAH rats and alleviated the cerebral edema. In addition, our study also found a significant increase in the expression level of GPR30 protein in hippocampal tissue of SAH rats, which may be to reduce the damage to the brain tissue of rats for protection. Moreover, the pathological histological examination showed that neurons in hippocampal tissue showed damage and loss, cell shrinkage, nuclei condensation, and concentrated and deep staining of nuclei and cytoplasm, and the administration of HT or G1 or G1 combined with HT significantly increased the GPR30 protein expression level in SAH rats and improved the pathological damage in hippocampal tissue. This finding is similar to the discoveries in previous studies. For instance, a study performed an intravenous injection of 300 μg/kg G1 1 h after SAH, and finally, the neurological function of male rats was improved, which could be explained by the reduction of TUNEL/FJC positive neuronal mortality in the brain tissue of male rats with SAH. Although the combined treatment of G1 and HT in this study shows a more pronounced intervention effect in SAH, further research into the mechanism of the combined treatment is still necessary.

A large number of literature studies suggested that those who have survived SAH will have varying degrees of medium‐long‐term neurocognitive impairment, which is primarily manifested as impaired balance, memory, and learning abilities, and impaired thought and expression (Turan et al., 2016). Multiple studies have demonstrated that the MWM test possesses various advantages, such as providing more experimental indexes to objectively reflect the rat's learning and memory abilities of spatial orientation (Zhong et al., 2017); NORT is a non‐aversion learning model depending on spontaneous exploration behavior of animals, which can detect the learning and memory abilities of animals (Okuda et al., 2004). Both the MWM test and NORT were adopted in this study to evaluate the learning and memory abilities of SAH rats. The results suggested that on the 4th and 28th days after operation, all SAH model groups with the longest escape latency had a significant decrease in the number of entries in the target quadrant and the time spent in the target quadrant of rats. However, compared to the SAH group, each treatment group with a lower escape latency, especially the SAH + G1 + HT group, significantly increased the number of entries in the target quadrant and the time spent in the target quadrant. It makes sense to assume that treatment of HT alone or G1 treatment alone can improve the learning and memory ability of SAH rats, but more importantly, the combined treatment appears to have a slightly better effect. Moreover, the anxiety‐ and emotion‐related behavior and social interaction of the rats were monitored using OFT, EPM test, three‐chamber paradigm test, and reciprocal social interaction test in this study. All test results demonstrated that, compared to the SAH group, the anxiety‐ and emotion‐related behavior of rats was reduced, and their social interaction enhanced obviously in all treatment groups, among which the SAH + G1 + HT group exhibited the strongest effect. It is speculated that this may be the result of the neuroprotective effect of G1 in the rats and the mitigative effect of HT therapy in brain tissue injuries.

## 5 CONCLUSION

Collectively, G1 combined with HT can improve neurological function and reduce the cerebral edema degree, thereby alleviating cognitive impairment and anxiety‐like behavior of SAH rats. The preliminary speculation of this result involves the respective functional effects of the two treatments. However, there are still some limitations in this study, like the lack of research into the mechanism of the combined treatment. Therefore, more exploration must be done on the detailed functional mechanism.

## AUTHOR CONTRIBUTIONS

Jun Peng and Zheng‐tao Yu were responsible for project design and conducting experiments, data analysis and interpretation, and manuscript and figure confection. Yang He conducted experiments, data analysis, and interpretation. Jun He provided technical support. Ji‐kun Zhang reviewed the manuscript and provided help with statistical analysis. Ying Xia conceived the project, interpreted data, provided technical and material support, and reviewed the manuscript. All authors have read and approved the submitted manuscript.

## CONFLICT OF INTEREST STATEMENT

The authors declare no conflicts of interest.

### PEER REVIEW

The peer review history for this article is available at https://publons.com/publon/10.1002/brb3.3204.

## Data Availability

The datasets used and analyzed in the current study are available from the corresponding author on reasonable request.
